# Nasal dorsal augmentation using diced cartilage with and without semi-circumferential fascia: technical note and retrospective monocentric study

**DOI:** 10.3389/fsurg.2025.1584561

**Published:** 2026-02-13

**Authors:** Laurence Pincet, Karma Lambercy, Florence Conty Lupascu, Philippe Pasche, Antoine Reinhard

**Affiliations:** 1 Department of Head and Neck Surgery, Centre Hospitalier Universitaire Vaudois, Lausanne, Switzerland; 2 Faculty of Biology and Medicine, University of Lausanne, Lausanne, Switzerland

**Keywords:** rhinoplasty, dorsal augmentation, diced cartilage, fascia graft, aesthetic surgery, patient satisfaction, surgical technique

## Abstract

**Introduction:**

Nasal dorsal augmentation is a fundamental step of rhinoplasty. It must provide height, projection, but also respect the aesthetic lines. Grafts made with diced-cartilage are moldable and have the capability to adequately adapt to the patient's anatomy. Many techniques have been described, with or without fascia wrapping.

**Objective:**

We describe two variations of the dorsal augmentation technique, using glued diced cartilage with and without semi-circumferential fascia. The cartilage is chopped, placed in a hemi syringe and covered with glue-tissue. A layer of fascia or perichondrium may be used to smooth the graft. It is still malleable and can be finely adjusted to the nose. We illustrate the technique and present the postoperative results; we used questionnaires to measure patients’ and surgeons’ satisfaction.

**Results:**

We included thirty-three patients, who underwent rhinoplasty with dorsal augmentation between September 2013 and January 2020. Nineteen were reconstructed with fascia, while fourteen, without. We chose the fascia technique mainly for women. There appeared to be greater satisfaction within the group with fascia, but not significant. Patients tended to be more satisfied if it was a first surgery rather than a revision, and if the origin of the deformity was anatomical, rather than post-traumatic or postoperative; women seemed more satisfied than men. There was no correlation between surgeons’ and patients’ satisfaction.

**Conclusion:**

The choice of surgical technique is made on a case-by-case basis, adapting to the patient's anatomy. We describe two techniques that are relatively simple, easily applicable and at the same time, provide regular and smooth grafts.

## Introduction

Reconstruction of the nasal dorsum during rhinoplasty is a delicate step for most surgeons. Patients and surgeons expect straightness, sufficient height, and regularity of the nasal surface. Numerous techniques have been described, from solid cartilage grafting—which may leave perceptible irregularities under the skin (1, 2)—to diced cartilage grafts, which risk dispersion and postoperative irregularities (1). In 1999, Erol described a technique using Surgicel to wrap diced cartilage, offering both malleability and volume (3). However, synthetic materials have fallen out of favor due to risks of chronic inflammation and variable resorption rates, making long-term results unpredictable (1, 4).

Consequently, many surgeons have turned to autologous fascia, such as temporal fascia, for improved outcomes (1, 4–6). Using autogenous diced cartilage remains the gold standard, offering moldability, adaptability, and a low complication profile (7–9). Wrapping diced cartilage in autologous fascia or perichondrium provides a smoother dorsal contour and may improve graft stability, especially in thin-skinned patients (10, 11).

Some authors suggest that perichondrium may offer superior chondrocyte viability compared to fascia (11). The principle remains the same: diced cartilage encased in a cylindrical structure made from sutured fascia or perichondrium. We have found that for minor irregularities, the fascia technique offers surface regularity without bulkiness, especially beneficial for patients with thin skin.

**Figure 1:**
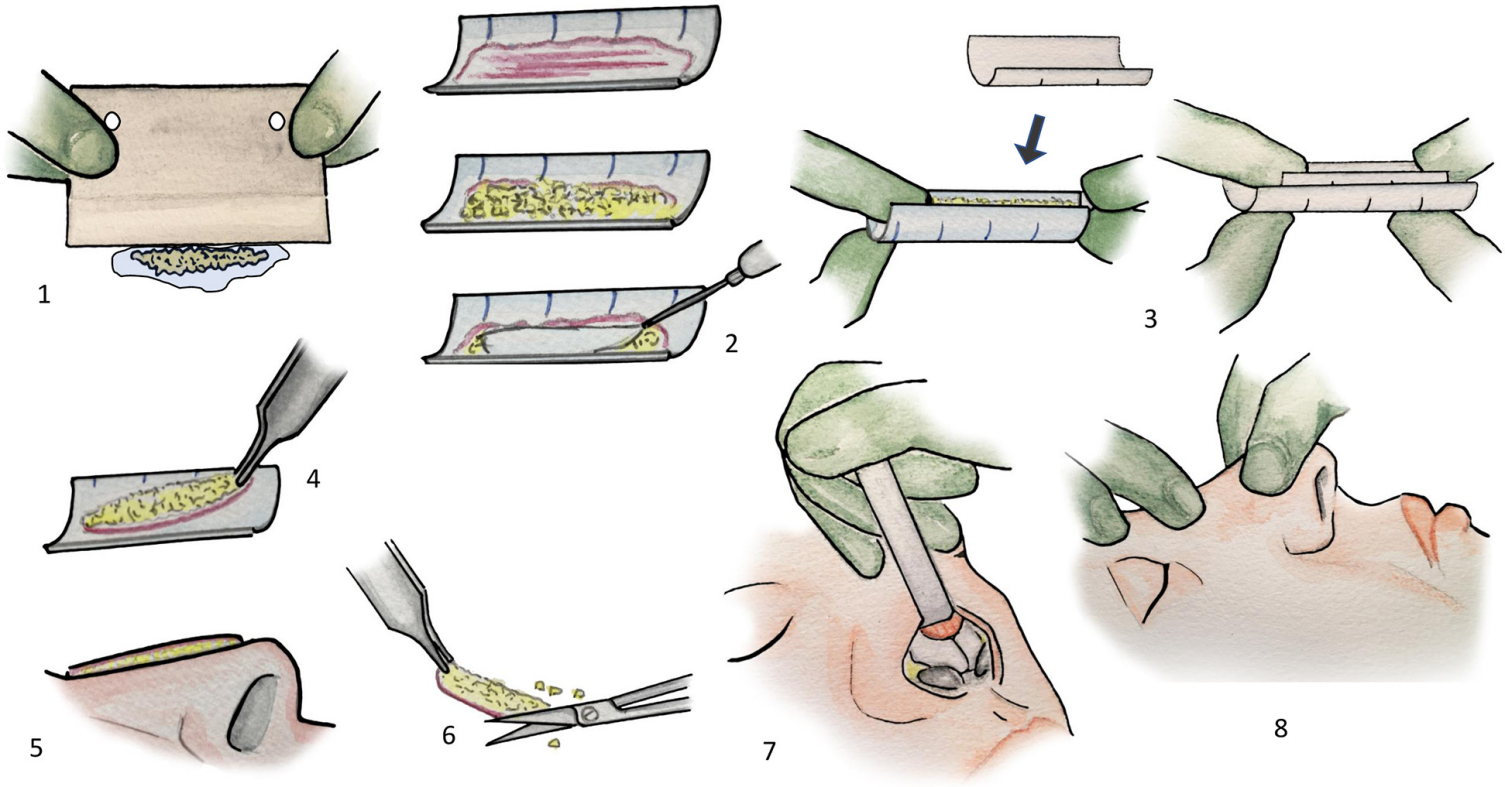
(1) The cartilage is finely chopped with a dermatome blade, and pieces are maintained together by capillarity in a drop of 0.9% NaCl. (2) The fascia or perichondrium is laid in a half 3 ml syringe. The diced cartilage and then the fibrin glue are delicately placed over. (3) With a half 1 ml syringe, a gentle pressure creates a slight depression in the semicylinder. (4) Once the graft is partially hardened, it can be handled. (5) The whole can be adapted to the patient's anatomy. (6) The graft is sculpted. (7) It is placed in the dorsum pocket. 8: The fibrin glue is still flexible and can be shaped once in place.

**Figure 2:**
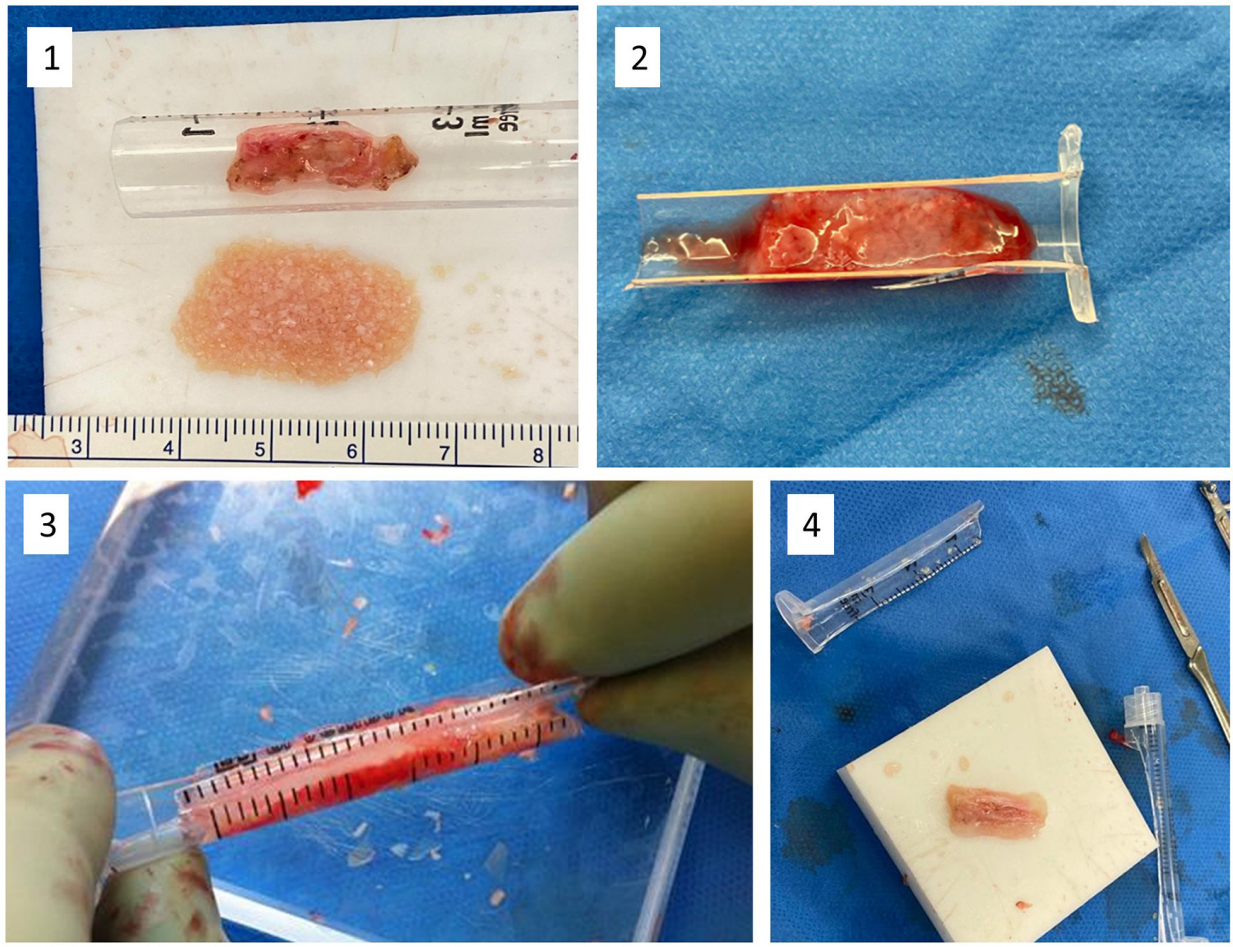
(1) Chopped cartilage and fascia in the syringe. (2) The chopped cartilage is placed on the fascia and covered with fibrin glue. (3) By a soft pressure with a half 3 ml syringe, we give a concave shape to the graft. (4) Once assembled, the graft is still malleable and can be sculpted.

In this study, we present two reproducible variations of dorsal augmentation using glued diced cartilage: one with and one without a semi-circumferential fascia layer. We detail our technical approach and assess outcomes through a retrospective study using validated and custom satisfaction questionnaires. Figures 1, 2 illustrate the surgical technique.

## Materials and methods

### Study design and patient selection

We conducted a retrospective monocentric study on all patients undergoing rhinoplasty with dorsal augmentation using diced cartilage with or without semi-circumferential fascia between September 2013 and January 2020. Ethics approval was obtained from the Commission d'Éthique Vaud (CER-VD Number 021-00113). Inclusion criteria: patients >15 years who underwent primary or revision rhinoplasty with our techniques. Exclusion: incomplete records or loss to follow-up.

### Surgical technique

The decision to use fascia was primarily guided by the patient's skin characteristics. In patients with thin or fragile skin—most frequently women—a fascia layer was preferred to enhance surface smoothness and reduce the risk of palpable irregularities. In contrast, fascia was used less frequently in men or in cases where skin thickness was deemed sufficient to mask minor asperities. The cartilage was harvested from a fragment of septal cartilage or an external site (rib or concha), depending on the volume of cartilage required. In addition, the choice of cartilage donor site could be influenced by the possibility of harvesting fascia from the same anatomical region. For instance, costal cartilage harvesting allowed the simultaneous collection of perichondrium through the same incision. Similarly, conchal cartilage was often combined with temporal fascia harvesting in moderate-volume cases. We used temporal fascia, muscular fascia of the rectus abdominis or pectoralis major muscle, or costal perichondrium.

As a binding agent, we used fibrin sealant (Tisseel®, Baxter). We finely chopped the cartilage using a dermatome blade with a drop of 0.9% NaCl on the cartilage. Small tilting movements of the blade—like chopping herbs—allowed us to increase the number of sections with greater precision than a standard scalpel blade (1). The drop of saline kept the fragments unified and eased the cutting process. After a few minutes of work, the diced cartilage measured less than 0.5 mm.

If a layer of fascia was used for interposition, we followed the steps illustrated in Figures 1, 2. We cut a 3 ml plastic syringe in half, lengthwise. The fascia was applied inside, then a drop of fibrin sealant was added, followed by the diced cartilage. Another few drops of fibrin sealant were layered on top. Gentle pressure using the other half of the syringe created a slight depression in the semi-cylinder. After approximately 2 min, once the glue had partially solidified, the graft could be retrieved, sculpted, and inserted into the nasal dorsum. The glue's gradual solidification allowed for intraoperative shaping of the graft to conform to the patient's anatomy while maintaining coherence for manipulation (2).

### Outcome measures

We used validated FACE-Q questionnaires (officially translated to French following back-translation and professional review) and a custom dorsum-specific questionnaire. Nasal function was not evaluated. Patients gave telephone consent before receiving questionnaires. Pre/post-operative photographs were evaluated by blinded surgeons using a surgeon-specific assessment form.

### Statistical analysis

We performed Mann–Whitney *U* tests and Spearman correlation for questionnaire analysis. Primary and revision cases were stratified. *P*-values < 0.05 were considered significant.

## Results

We contacted 64 patients; 33 responded (52%): 14 fascia, 19 no fascia. Mean age: 33 years (range: 15–58). Women were more likely to receive fascia grafts. Nineteen primary and 14 revision rhinoplasties were included. Average follow-up: 52 months (Table 1).

**Table 1:** Patients’ characteristics.

Results	With fascia	Without fascia	Total
Number of patients	14	19	**33**
Men/Women	6/8	15/4	**21/12**
Age at the time of surgery: mean (range)	36 (18–48)	30 (15–58)	**33 (15–58)**
Follow-up: mean (range)	54 (12.5–85)	49 (15.5–86)	**52 (12.5–86)**
Origin of the deformity:			
Traumatic	8	9	**17 (52%)**
Post-operative	2	3	**5 (15%)**
Wegener	0	1	**1 (3%)**
Anatomical	4	6	**10 (31%)**
Cartilage graft taking:			
Costal,	6	7	**13 (40%)**
Septal,	1	10	**11 (33%)**
Conch	7	2	**9 (27%)**
Number of previous surgeries:			
1st surgery	8	11	**19 (58%)**
2nd surgery	3	7	**10 (31%)**
3rd surgery	1	0	**1 (3%)**
4th surgery	1	0	**1 (3%)**
>4 surgeries	1	1	**2 (6%)**
Associated rhinologic procedure:			
Septoplasty,	12	16	**28**
Perforation Closure,	1	1	**2**
Spreader Graft,	7	10	**17**
Batten graft,	2	7	**9**
Collumellar struts,	3	9	**12**
Rim graft,	3	0	**3**
Shield graft,	2	1	**3**
Cap graft,	1	1	**2**
Tongue-in-groove,	1	1	**2**
Sheen Graft	0	1	**1**

Septal cartilage was used preferentially; costal and conchal when needed. Costal cartilage was associated with more postoperative discomfort (*p* = 0.01); no significant difference in scar-related complaints.

No significant difference in satisfaction scores between groups. Women showed slightly higher satisfaction, especially regarding revision desire. Primary cases and anatomical deformities yielded better outcomes.

Surgeon assessments showed moderate inter-rater correlation (*ρ* = 0.66) but poor correlation with patient satisfaction. One patient developed tip inflammation (fascia group), resolved with antibiotics. One pleural breach occurred during rib harvest, no pneumothorax. No revisions were needed.

**Figure 3:**
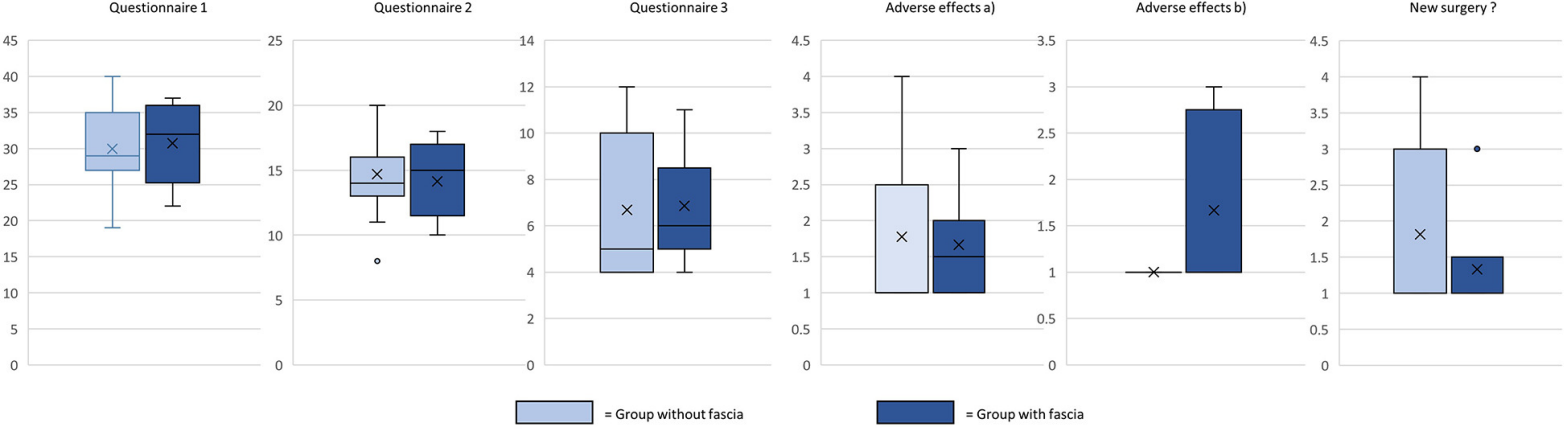
Response to satisfaction questionnaires, box plots.

**Figure 4:**
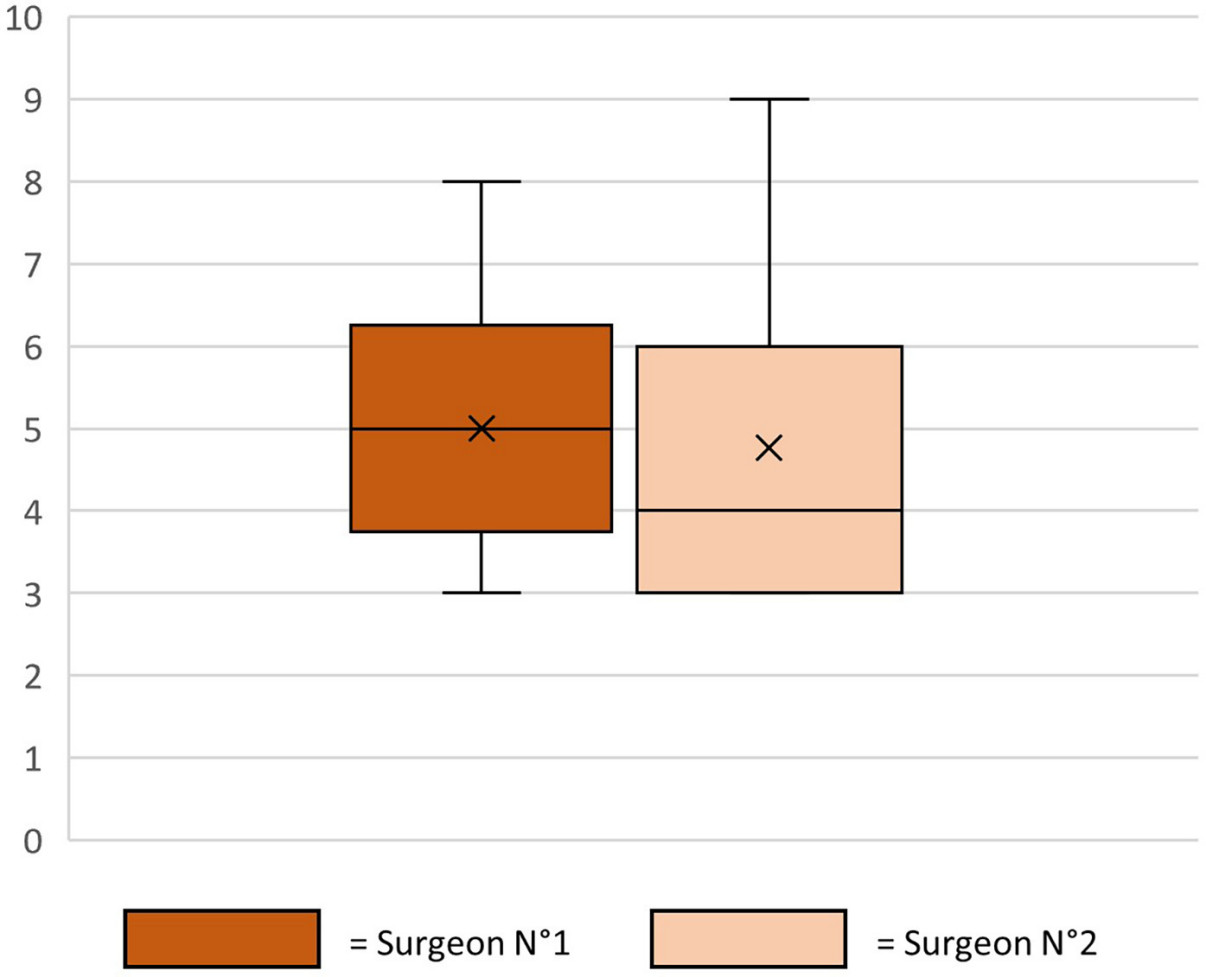
Evaluation of patient photographs by two independent surgeons.

**Figure 5:**
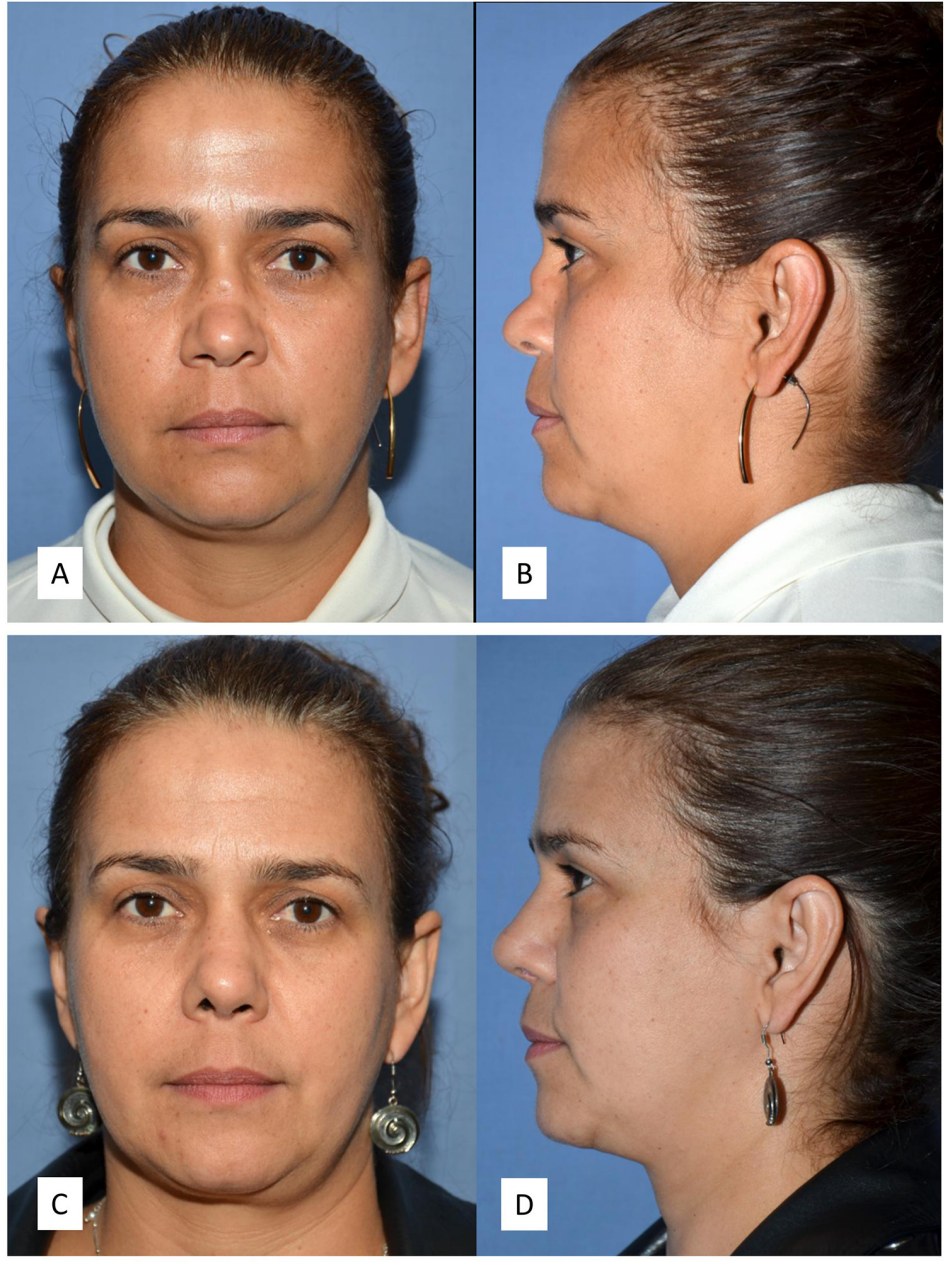
Rhinoplasty for a significant dorsum augmentation, for a patient with chronic atrophic polychondritis. The graft was covered with fascia. The procedure also included bilateral spreader grafts, a septal extension graft and a shield graft. **(A**,**B)** Preoperative front and side photos. **(C**,**D)** Postoperative front and side photos.

**Figure 6:**
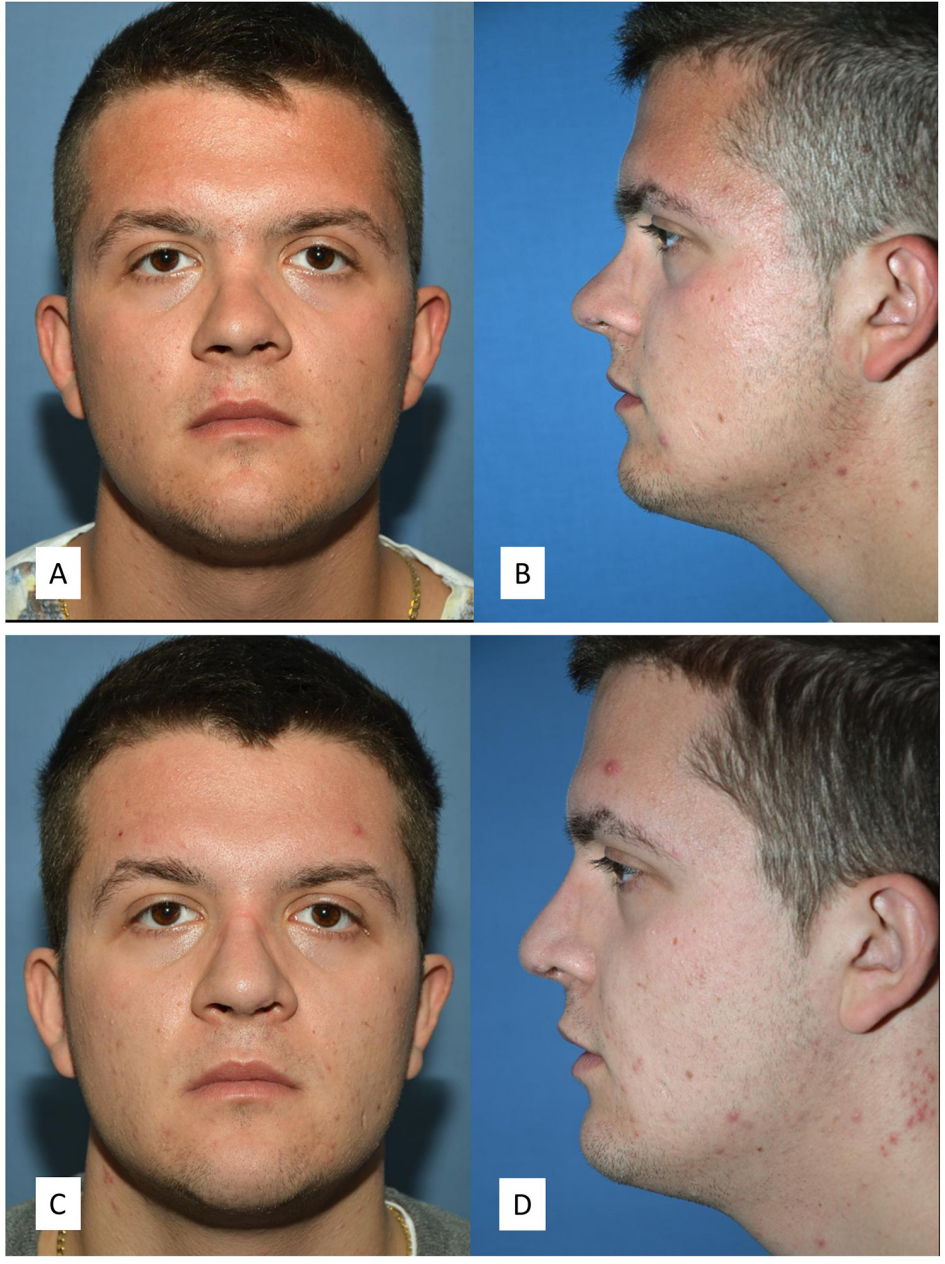
Rhinoplasty with dorsum augmentation after nasal and septal fracture. The diced cartilage graft was without fascia. The procedure also included an extracorporeal septoplasty, bilateral spreader grafts, and a columellar strut. **(A**,**B)** Preoperative front and side photos. **(C**,**D)** Postoperative front and side photos.

Patient responses to questionnaires are summarized in Table 2; Figure 3. Surgeon assessments are illustrated in Figure 4, with statistical correlation results in Table 3. Pre- and postoperative results are shown in Figures 5, 6.

**Table 2:** Patients' satisfaction outcomes according to surgical technique (with or without fascia), sex, type of surgery, and etiology of the deformity.

Questionnaire	*P* value
Overall patients, patients are more satisfied in the group with fascia than in the group without fascia:	
Q1	>0.05
Q2	>0.05
Q3	=0.2
Do you think that you need a new surgery?	=0.2
Among women, women are more satisfied in the group with fascia than in the group without fascia:	
Q1	>0.05
Q2	>0.05
Q3	>0.05
Do you think that you need a new surgery?	>0.05
Among men, men more satisfied in the group with fascia than in the group without fascia:	
Q1	>0.05
Q2	>0.05
Q3	>0.05
Do you think that you need a new surgery?	>0.05
Patients are more satisfied after a first surgery that after a revision surgery:	
Q1	>0.05
Q2	>0.05
Q3	>0.05
Do you think that you need a new surgery?	>0.05
Women are more satisfied than men:	
Q1	>0.05
Q2	>0.05
Q3	>0.05
Do you think that you need a new surgery?	=0.05
Concerning the tolerance of the donor site: patient are more complaining about the is costal harvest site than concha:	
Aesthetic aspect of the scar?	=0.1
Persistent pain?	=0.01
Patient are more satisfied if the nose deformation is anatomical than the sequelae of a previous surgery or post-traumatic:	
Q1	=0.05
Q2	=0.1
Q3	<0.05
Adverse effect a	<0.05
Adverse effect b	>0.05
Need for revision surgery?	<0.05

**Table 3:** Spearman correlation between surgeons’ assesment and patients’ assesment.

Correlation beetween		Spearman correlation
Surgeon N°1	Q1	−0.2861538
	Q2	−0.042735
	New surgery?	0.39418803
Surgeon N°2	Q1	−0.1254701
	Q2	−0.0208547
	New surgery?	0.27111111
Surgeon N°1	Surgeon N°2	0.65880342

## Discussion

Surgery of the nasal dorsum is delicate, and patients are critical of the slightest anomaly or asperity (12). We propose a detailed description of two techniques and their outcomes.

Hemi-cylinder diced cartilage without fascia:

This method reduces operative time and avoids donor site morbidity, especially when the cartilage is taken from the nasal septum. Free diced cartilage has been used, but we prefer glued cartilage to prevent dispersion (2). Adhesion to skin and loss of gliding plane are potential downsides, particularly in thin-skinned patients. In order to avoid feeling the irregularities of diced cartilage, especially in cases of thin skin, it is recommended to use ultra-diced cartilage (<0.5 mm) (add reference).

### Hemi-cylinder diced cartilage with fascia

For significant volume loss or fragile skin, the fascia layer improves smoothness and gliding. Our method simplifies previous fascia-cylinder techniques, avoiding suturing while retaining modelability. Compared to Erol's closed-cylinder technique (3), ours allows for finer adjustment, reduces bulk and decreases operating time.

### Cartilage source selection

Septal cartilage is preferred when feasible. Conchal cartilage with temporal fascia is suitable for moderate needs, while costal cartilage offers larger volumes. Perichondrium or local fascia can be harvested through the same incision during rib grafting. Crushing cartilage is contraindicated as it compromises chondrocyte viability and graft success (13).

### Binding agents

We used fibrin glue for ease and robustness. Alternatives such as autologous blood glue, PRP, or CGF may offer cellular benefits in animal models (1), but have not shown superiority in aesthetic outcomes in human studies.

Our study found higher satisfaction in women, primary surgeries, and anatomical deformities. Surgeon-patient satisfaction correlation was low, reflecting the subjective nature of aesthetic outcomes. SIMON/SYLVIA criteria may help predict satisfaction (14, 15). Biases include selection bias, small cohort, and imbalance between groups.

While not aiming to prove superiority, this study highlights the utility of fascia in specific cases. Our technique with fascia appears particularly useful for smoothing minor asperities and improving contour in patients with thin skin.

Emerging techniques such as fat injection may complement traditional grafts. We have begun integrating this in select cases with promising preliminary results (16–18).

## Conclusion

Dorsal augmentation techniques have evolved significantly. Our experience with diced cartilage semi-cylinders—either alone or fascia-covered—demonstrates reproducible, adaptable, and aesthetically satisfying outcomes. The fascia technique, easier to perform than classic fascia wraps, may offer additional benefits in contour refinement. Technique selection should be tailored to the individual patient.

## Data Availability

The original contributions presented in the study are included in the article/Supplementary Material, further inquiries can be directed to the corresponding author.
