# Supplementary material for: Nasal dorsal augmentation using diced cartilage with and without semi-circumferential fascia: technical note and retrospective monocentric study
Source: Front Surg. 2026 Feb 13;12:1584561. doi: 10.3389/fsurg.2025.1584561 (PMC12947388; doi:10.3389/fsurg.2025.1584561)
Supplement: Supplementary file 1 [file Datasheet1.docx]

**Questionnaire 1 :** FACE-QTM – SATISFACTION WITH NOSE

Copyright©2013 Memorial Sloan Kettering Cancer Center, New York, USA. All rights reserved.

**Questionnaire 2 :**

For each question, circle only one answer. How satisfied are you with:

|  | Very unsatisfied | Very unsatisfied | | Quite satisfied | Very satisfied |
| --- | --- | --- | --- | --- | --- |
| a. The texture of the dorsum to the touch? | **1** | **2** | **3** | | **4** |
| b. the regularity of the dorsum? | **1** | **2** | **3** | | **4** |
| c. From the front, how straight is your nose? | **1** | **2** | **3** | | **4** |
| d. Seen from the side, how do you estimate the height of the nose? | **1** | **2** | **3** | | **4** |
| e. How would you rate the overall harmony of your nose? | **1** | **2** | **3** | | **4** |

**Questionnaire 3 :** FACE-QTM - ADVERSE EFFECTS: NOSE

Copyright©2013 Memorial Sloan Kettering Cancer Center, New York, USA. All rights reserved.

**TO FINISH :**

You have had a graft taken from the ear and behind the ear / a rib. How disturbed are you by the cartilage harvesting site:

|  | Not at all | A little | Medium | A lot |
| --- | --- | --- | --- | --- |
| a. Aesthetic aspect of the scar ? | **1** | **2** | **3** | **4** |
| b. Persistent pain? | **1** | **2** | **3** | **4** |

|  | Not at all | A little | Medium | A lot |
| --- | --- | --- | --- | --- |
| **Do you feel that you need a new operation?** | **1** | **2** | **3** | **4** |
