# Supplementary material for: Nasal dorsal augmentation using diced cartilage with and without semi-circumferential fascia: technical note and retrospective monocentric study
Source: Front Surg. 2026 Feb 13;12:1584561. doi: 10.3389/fsurg.2025.1584561 (PMC12947388; doi:10.3389/fsurg.2025.1584561)
Supplement: Supplementary file 2 [file Datasheet2.docx]

Patient code:

Surgeon n° :

**What do you think of :**

|  | Very good | Good | Medium | Bad |
| --- | --- | --- | --- | --- |
| a. In profile, the persistence of hump or indentation? | **1** | **2** | **3** | **4** |
| b. In profile, the height of the dorsum | **1** | **2** | **3** | **4** |
| c. From the front, the straightness of the nose | **1** | **2** | **3** | **4** |

**Total : / 12**

| .............................. | .............................. ........................ |
| --- | --- |
| Place and date | Signature |
